# Grounding context in face processing: color, emotion, and gender

**DOI:** 10.3389/fpsyg.2015.00322

**Published:** 2015-03-24

**Authors:** Sandrine Gil, Ludovic Le Bigot

**Affiliations:** University of Poitiers and CNRS (UMR 7295 – Centre de Recherches sur la Cognition et l’Apprentissage), PoitiersFrance

**Keywords:** emotion, color, facial expression, gender, implicit affective processing

## Abstract

In recent years, researchers have become interested in the way that the affective quality of contextual information transfers to a perceived target. We therefore examined the effect of a red (vs. green, mixed red/green, and achromatic) background – known to be valenced – on the processing of stimuli that play a key role in human interactions, namely facial expressions. We also examined whether the valenced-color effect can be modulated by gender, which is also known to be valenced. Female and male adult participants performed a categorization task of facial expressions of emotion in which the faces of female and male posers expressing two ambiguous emotions (i.e., neutral and surprise) were presented against the four different colored backgrounds. Additionally, this task was completed by collecting subjective ratings for each colored background in the form of five semantic differential scales corresponding to both discrete and dimensional perspectives of emotion. We found that the red background resulted in more negative face perception than the green background, whether the poser was female or male. However, whereas this valenced-color effect was the only effect for female posers, for male posers, the effect was modulated by both the nature of the ambiguous emotion and the decoder’s gender. Overall, our findings offer evidence that color and gender have a common valence-based dimension.

## Introduction

The idea that color can have affective meaning, and therefore influences psychological functioning, is certainly not novel (e.g., Goldstein, 1942), but was elaborated upon in several recent studies (e.g., Elliot and Maier, 2007). In this context, numerous studies have been conducted on the color red, which probably derives its strength of meaning from both evolution and experience. Even though the color-in-context theory developed by Elliot and Maier (2007) suggests that the same color can have different meanings in different contexts (e.g., Elliot and Niesta, 2008; Meier et al., 2012), red has been extensively associated with negative meaning, supported by two lines of research. First, when humans are exposed to the color red in an achievement context, their ability to solve problems seems to be significantly hampered, compared with when they are exposed to another color (for reviews, see Elliot and Maier, 2012, 2014). The second–complementary–line of research has directly demonstrated the red-negativity association, using congruency/interference effects. For instance, a negative emotional target is more accurately and rapidly processed (Moller et al., 2009), more convincing (Gerend and Sias, 2009), and more salient, and thus better memorized (Kuhbandner and Pekrun, 2013) when it is perceived in a context (e.g., red) that evokes the same emotion. This increasingly abundant literature, inspired by pioneer study of Valdez and Mehrabian (1994), indicates that color can convey a specific meaning, namely emotion (e.g., Suk and Irtel, 2010; Simmons, 2011).

In real life, the faces we encounter are embedded in a natural plethora of information sources, and a growing body of literature has looked at emotional face processing in a wide range of emotional contexts (for reviews, see Barrett et al., 2011; Wieser and Brosch, 2012). Within this framework, color can be regarded as one such source of contextual information, and thus as an *implicit affective cue* (Friedman and Förster, 2010). Researchers have therefore recently examined how color is subjectively associated with emotional faces (Palmer et al., 2013) or used as an implicit source of contextual information for faces (Frühholz et al., 2011; Young et al., 2013; Gil and Le Bigot, 2014). Regarding red, Palmer et al. (2013), Experiment 2 found that when participants had to choose the color (out of possible 37) that was the most/least consistent with an emotional face, angry faces were particularly closely associated with reddish colors. In Young et al.’s (2013) study, results showed that, compared with a green or achromatic priming, a red priming facilitated the categorization of angry as opposed to happy faces.

When humans process an emotional face, the information communicated by that face is considered in what we can call a *grounding context*, which goes beyond the perceptual context *per se*, and relates to the different forms of intention that are attributed to the encoder, as well as to the inherent characteristics of the decoder (Schwarz et al., 2013). This grounding context can only be properly considered if we acknowledge that emotion is not a gender-neutral concept. First, differences in emotion processing are known to exist between men and women with, for instance, women exhibiting more intense reactions to aversive stimuli (e.g., Schienle et al., 2005). Second, findings suggest that gender differences may be exacerbated by general gender stereotypes: stereotypes create expectations, and also roles and display rules. As a result, happiness is detected faster in female faces, and anger faster in male faces (e.g., Öhman et al., 2010), while the male gender generally evokes more negative emotions (e.g., Hess et al., 1997; Brebner, 2003; Fischer et al., 2004). Despite this evidence that gender is a crucial factor in emotion processing, reports usually either do not mention this factor or else use it solely as a control variable (i.e., with female stimuli and female participants), probably because of the complexity of doing otherwise.

To our knowledge, few color studies have so far examined the effect of gender when considering color-meaning associations. Regarding color as a context for face processing, Young et al. (2013, p. 383) recently acknowledged that “future work varying target sex as a factor could be theoretically and practically valuable.” Therefore, in order to deepen our understanding of how different valenced contextual features work together in face processing, our study included three potential types of valenced contextual information: color, poser’s gender, and decoder’s gender.

We administered a categorization task in which faces were embedded in colored backgrounds. In everyday life, people are called upon to process a wide range of emotional facial expressions, many of which are far less intense and distinct than prototypical ones (e.g., Motley and Camden, 1988; Coren and Russell, 1992). We therefore examined whether color can bias processing of ambiguous emotional faces, that is, faces that are considered to be particularly influenced by context. Participants were shown two kinds of faces displaying either a neutral emotion–the least distinctive emotion *by definition* (e.g., Carrera-Levillain and Fernandez-Dols, 1994; Cooney et al., 2006)–or surprise, which is regarded as ambiguous because it can signal either a positive or a negative unexpected event, and because it has features in common with expressions of happiness (i.e., open mouth), but also fear (i.e., open eyes; e.g., Adolphs, 2002; Kim et al., 2004). This categorization of facial expressions of emotion therefore constituted an implicit color-meaning investigation, and was supplemented by an explicit task in the form of subjective color ratings, as a follow-up from previous studies featuring self-report measures (e.g., Hemphill, 1996; Palmer et al., 2013).

The first goal of the present study was to examine whether the color-emotion association can bias the processing of an ambiguous target. We predicted that red would disambiguate expressions in a negative direction, compared with control background colors, and above all compared with green, given that several studies have suggested that red and green are opposite-valenced colors (e.g., Moller et al., 2009; Kuhbandner and Pekrun, 2013).

Since female and male participants were shown both male and female faces, the second goal of our study was to explore the effect of gender valence on the color-emotion association. We expected the impact of red on face processing to interact with both the poser’s and the decoder’s gender, notably enhancing the negativity of the male-related negative valence.

## Materials and Methods

### Participants

Participants were 24 female (*M* = 19.4 years, SD = 1.22) and 20 male French students (*M* = 21.7, SD = 1.98), who took part in exchange for course credits. They all provided their written informed consent to participate in this study, which was conducted in accordance with the Declaration of Helsinki. Participants had normal or corrected-to-normal visual acuity, and normal color vision, as assessed with the short form (i.e., six plates) of the Ishihara Color Vision Test (Ishihara, 1917/2009).

### Material

The experiment was controlled by a PC using E-Prime 1.2 software (Psychology Software Tools, Pittsburg, PA, USA). The “D” and “K” keys on the keyboard were used for the responses.

The initial stimulus set comprised front-view images of the faces of 52 different posers-half women, half men-taken from the Karolinska Directed Emotional Faces (KDEF) database (Lundqvist et al., 1998). Each poser expressed a neutral and a surprised expression, and each image was displayed in the middle of the screen against four different color backgrounds. The final set consisted of 416 stimuli (52 × 2 × 4).

Two of the backgrounds featured colors with theoretically opposed meanings: red and green (e.g., Elliot et al., 2007; Moller et al., 2009; Kuhbandner and Pekrun, 2013). The other two backgrounds were used as control: one was achromatic, as in previous studies (e.g., Elliot et al., 2007, 2009), while the other combined the two hypothetically opposed colors (i.e., 50% red and 50% green: mixed red/green background). All four color backgrounds were created according to the hue, saturation, and lightness (HSL) system. There was full (100%) saturation. To control lightness, and solve the problem of matching chromatic and achromatic colors (i.e., the question of whether an achromatic color can be a *good* control), the backgrounds consisted of randomly configured patchworks of colors of 11 different lightnesses (i.e., 50–100%), with an equivalence between the chromatic and achromatic backgrounds, except for hue. Hue was 0° for red and 120° for green on the color wheel. It should be noted that we based the characterization of colors on the HSL system, and did not use a spectrophotometer.

In addition to the main task, participants were asked to rate the background colors on five 9-point Osgood scales, from both discrete and dimensional perspectives of emotion: *Peur* (Fear) vs. *Colère* (Anger), *Tristesse* (Sadness) vs. *Joie* (Happiness), *Négatif* (Negative) vs. *Positif* (Positive; i.e., Valence), *Tranquillité* (Calm) vs. *Excitation* (Arousing; i.e., Arousal), and *Attractive* (Attractiveness) vs. *Non-attractive* (Unattractiveness). For the purpose of these ratings, participants were presented with an image comprising the four color backgrounds labeled with the letters A–D.

### Procedure

**FIGURE 1:**
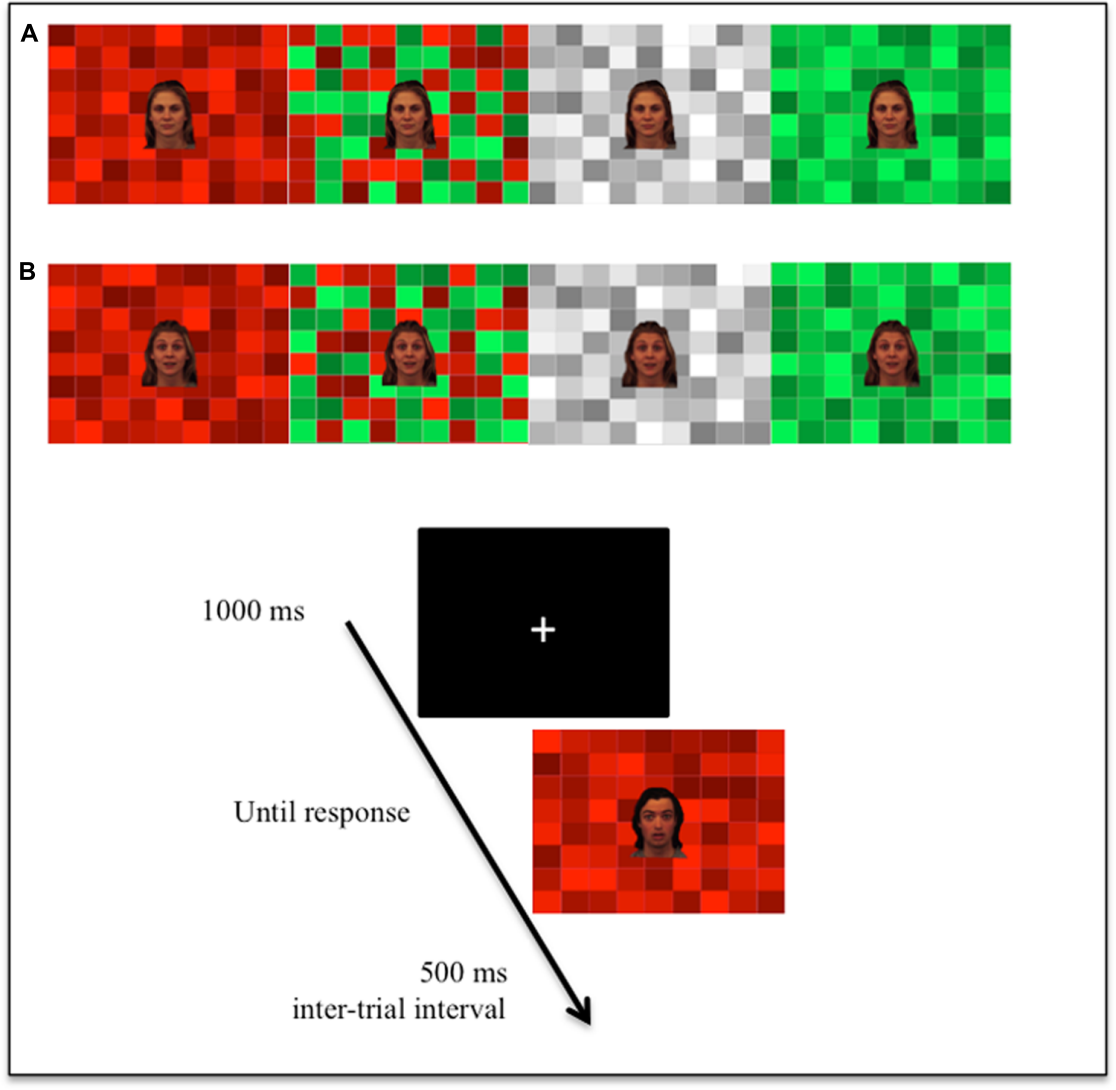
**Illustration of the stimuli and experimental design. (A)** Example of one female face expressing the two emotions (neutral vs. surprise) against the four different background colors (red, mixed, achromatic, and green). **(B)** Illustration of a trial, with an example of a male face expressing surprise.

Participants were tested one at a time in the laboratory, where they were seated in front of the computer, 60 cm from the screen. After being told that the experiment concerned the categorization of ambiguous emotional expressions, participants performed 32 practice trials. They were instructed to indicate whether the faces expressed broadly negative or broadly positive emotions, by pressing the key corresponding to their response as quickly as possible (the keys were counterbalanced across participants). Following the practice trials, participants performed the test phase in the absence of the experimenter. A total of 384 trials were administered in random order, divided into four blocks (i.e., 96 × 4). These blocks were separated by short breaks of 30 s. Each trial began with a fixation cross (1000 ms), followed by the onset of the stimulus, which disappeared when the participant gave a response (see **Figure 1**). The intertrial interval was 500 ms. We recorded the participants’ positive/negative responses and the corresponding reaction times for all the trials. Finally, and in the presence of the experimenter, participants assessed each color on the different emotional dimensions, placing the corresponding letters on the five Osgood rating scales. The presentation order of the images (displayed on the monitor’s) and the scales (printed on sheets of paper) was randomized across participants.

## Results

### Categorization Task

#### Statistical Analyses

Analyses were performed with SAS Version 9.4 statistical software (SAS Institute Inc., Cary, NC, USA; GLIMMIX procedure). As the outcome variable was binary (i.e., negative vs. positive responses), we used logistic mixed models to analyze the negative responses (see Baayen et al., 2008; Jaeger, 2008; Barr, 2013). Linear mixed models were used to analyze reaction times for the negative responses. Reaction times below 250 ms or above 3000 ms were treated as outliers, and the corresponding trials (2% of all trials) were removed from our dataset for both response and reaction time analyses. As analysis of reaction times failed to reveal either main or interaction effects of color, we only report results for the negative responses. Finally, to examine in greater detail how color, poser’s gender, and decoder’s gender interacted in target processing–and in order to report more meaningful analyses–we analyzed the data for female and male posers separately (for details of the statistical models, see Supplementary Material – Table 1 for female faces analyses, and Table 2 for male faces analyses).

#### Female Face Analysis

**FIGURE 2:**
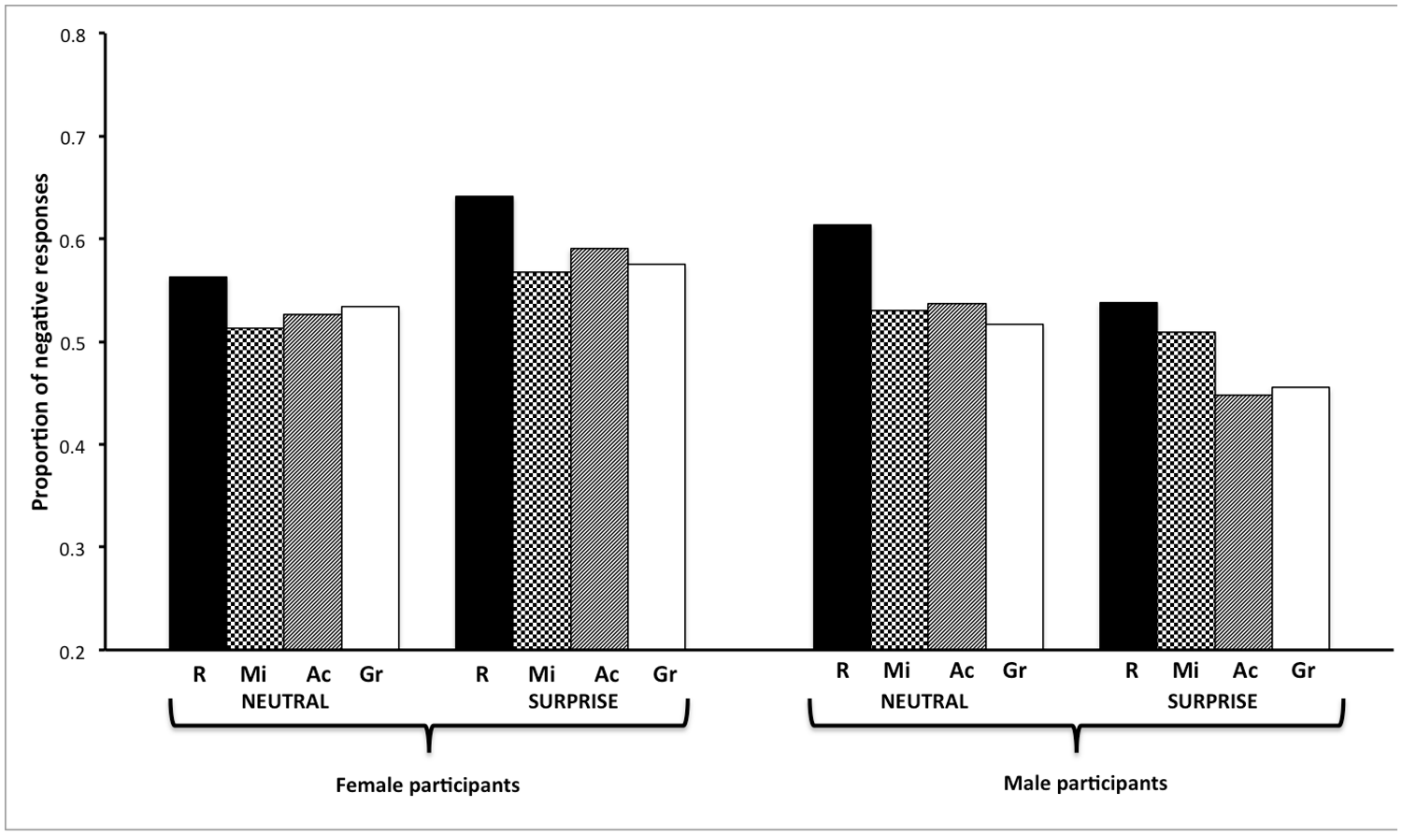
**Mean proportion of negative responses to female faces, according to background color (Ac, achromatic; Gr, green; Mi, mixed; R, red), displayed emotion (neutral vs. surprise), and decoder gender (female vs. male)**.

As illustrated in **Figure 2**, the red background was more likely to prompt negative responses than the green [*OR* = 0.68, 95% CI (0.56,0.82)], achromatic [*OR* = 0.70, 95% CI (0.58,0.85)], or mixed [*OR* = 0.71, 95% CI (0.59,0.86)] backgrounds, which did not differ from each other. Analysis showed that only color significantly predicted negative responses, *F*(3,128.1) = 7.20, *p* = 0.0002. Neither emotion, *F*(1,53.97) = 0.04, *p* = 0.84, nor decoder’s gender, *F*(1,93.42) = 1.03, *p* = 0.31, predicted negative responses. In addition, analysis showed that none of the interactions involving these factors were significant (all *p*s > 0.1).

#### Male Face Analysis

**FIGURE 3:**
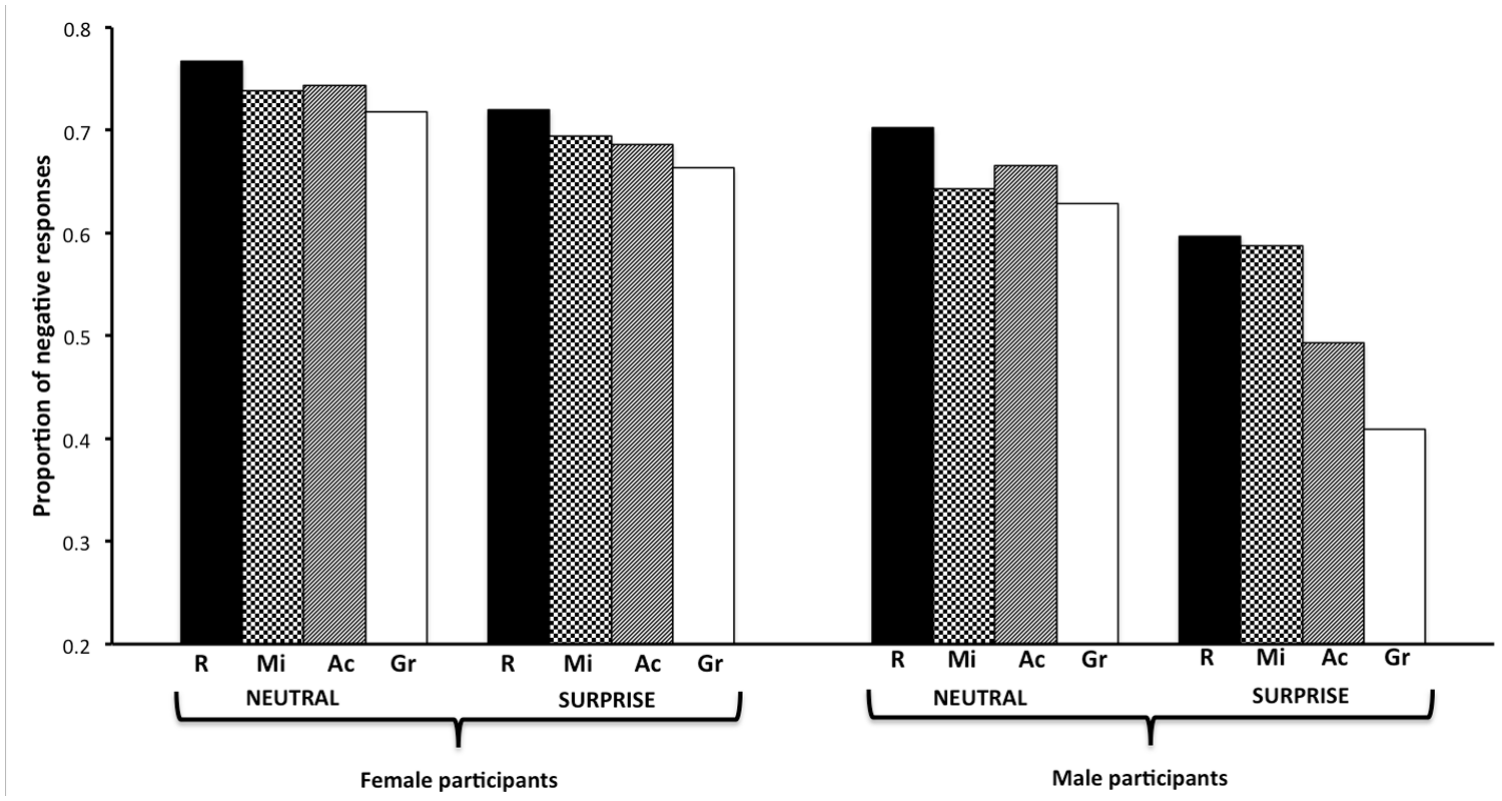
**Mean proportion of negative responses to male faces, depending on background color (Ac, achromatic; Gr, green; Mi, mixed; R, red), displayed emotion (neutral vs. surprise), and decoder gender (female vs. male)**.

As illustrated in **Figure 3**, and as for female faces, the red background was more likely to prompt negative responses than either the green [*OR* = 0.58, 95% CI = (0.48,0.71)], achromatic [*OR* = 0.75, 95% CI (0.62,0.92)], or mixed [*OR* = 0.82, 95% CI (0.68,1)] backgrounds. Moreover, neutral faces were more likely to elicit negative responses than surprised ones [*OR* = 1.95, 95% CI (1.01,3.76)], and female participants were more likely to give negative responses than male participants [*OR* = 0.46, 95% CI (0.28,0.75)]. Thus, statistical analysis showed that color, emotion, and decoder’s gender significantly predicted negative responses, *F*(3,136.9) = 10.25, *p* < 0.0001, *F*(1,51.9) = 4.19, *p* = 0.04, and *F*(1,40.3) = 10.44, *p* = 0.002, respectively.

Regarding the notion of grounding context, theses different variables appeared to have a modulating effect on negative responses. Indeed, the Color × Emotion, and Color × Emotion × Decoder’s gender interactions were both significant, *F*(3,8164) = 3.33, *p* = 0.02, and *F*(3,8164) = 3.00, *p* = 0.03, respectively. It is worth noting that the red background elicited (or tended to elicit) more negative responses than the green one for both neutral (*p* = 0.07) and surprised faces (*p* < 0.0001). However, the second-order interaction indicated that whereas the red background was indeed likely to prompt more negative responses than the other colors, extent to which it did so depended on both the emotional face and the decoder’s gender. **Figure 3** shows that neutral faces were likely to elicit more negative responses than surprised ones, but that the extent of this difference depended on the nature of the color background, especially when the decoder was male. In other words, this second-order interaction resulted largely from the fact that female participants gave a high proportion of negative responses across all conditions (66–77%), whereas male participants gave fewer negative responses (40–70%), especially for surprised faces (40–60%). Accordingly, the extent to which negative responses differed as a function of background color depended on these two factors.

### Subjective Color Ratings

**FIGURE 4:**
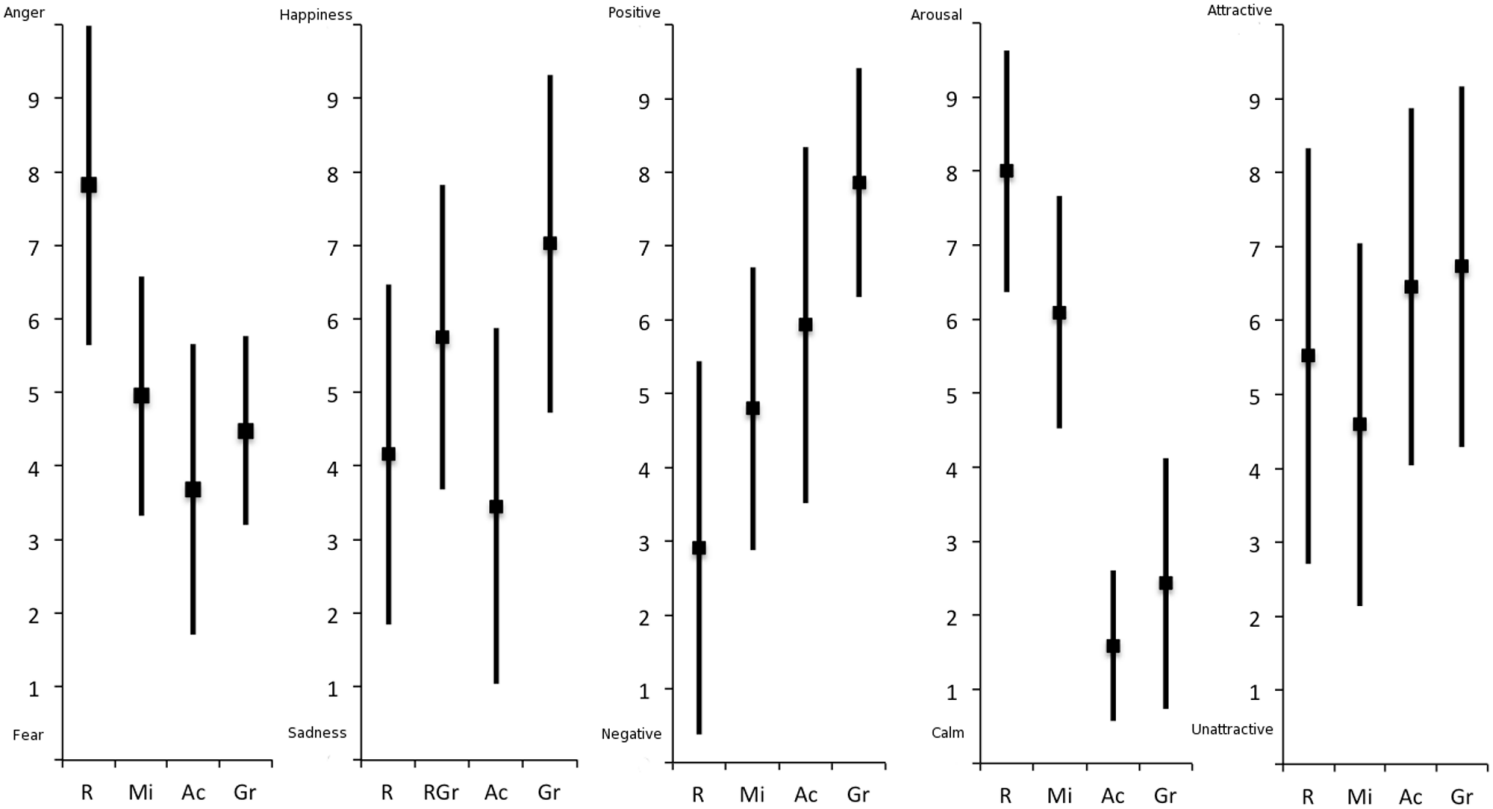
**Mean (SD) color ratings.** From left to right: fear vs. anger, sadness vs. happiness, negative vs. positive, calm vs. arousal, unattractive vs. attractive. R, red; Ac, achromatic; Mi, mixed green/red; Gr, green.

The bipolar Osgood scales allowed us to see how participants subjectively assessed each color background on five affective scales. As Mann-Whitney analyses failed to reveal a significant effect of decoder’s gender for any dimension, the analyses reported below were run on data for both genders. We subjected the ratings (illustrated in **Figure 4**) to non-parametric tests (i.e., Friedman’s analysis of variance and the Wilcoxon signed-rank test for pairwise comparisons).

Color had a significant effect on Fear–Anger ratings, chi_F_^2^(3) = 56.29, *p* < 0.001, with red being judged to be significantly closer to anger (*M* = 7.82, SD = 2.17; all *p*s < 0.001). Conversely, the achromatic background was judged to be significantly closer to fear (*M* = 3.68, SD = 1.97). The green (*M* = 4.48, SD = 1.28) and mixed (*M* = 4.95, SD = 1.63) backgrounds were not judged to be close either to fear or to anger, and did not differ significantly from each other (*p* = 0.12).

Analysis also revealed a significant effect of color on Sadness-Happiness ratings, chi_F_^2^(3) = 37.80, *p* < 0.001. Green was rated as the happiest color (*M* = 7.02, SD = 2.30), and differed significantly from all the other background colors (all *p*s < 0.001). Green was followed by the mixed background (*M* = 5.75, SD = 2.07), which differed from both the achromatic (*M* = 3.45, SD = 2.42), and red (*M* = 4.16, SD = 2.31) backgrounds. The achromatic and red backgrounds were therefore judged to be closest to sadness, and did not differ significantly from each other (*p* = 0.27).

There was a significant effect of color on Valence ratings, chi_F_^2^(3) = 60.02, *p* < 0.001. Red was judged to be the most negative color (*M* = 2.91, SD = 2.53) and green the most positive one (*M* = 7.86, SD = 1.55), with the achromatic (*M* = 5.93, SD = 2.42), and mixed (*M* = 4.79, SD = 1.91) backgrounds coming in between. All pairwise comparisons were significant (all *p*s < 0.05).

There was a significant effect of color on Arousal ratings, chi_F_^2^(3) = 99.55, *p* < 0.001, where again all pairwise comparisons were significant (all *p*s < 0.05). More specifically, red was rated as the most arousing color (*M* = 8, SD = 1.63), followed by the mixed background (*M* = 6.09, SD = 1.57). Conversely, the achromatic background was rated as the least arousing one (*M* = 1.59, SD = 1.02), followed by green (*M* = 2.43, SD = 1.69).

Last, analysis showed a significant effect of color on Attractiveness ratings, chi_F_^2^(3) = 13.57, *p* = 0.004, even if none of the colors seemed particularly attractive to participants (all *M*s < 7). The mixed (*M* = 4.59, SD = 2.45) background was judged to be the least attractive, differing significantly from the achromatic (*M* = 6.45, SD = 2.42), and green (*M* = 6.72, SD = 2.44) backgrounds, but not from the red one (*M* = 5.52, SD = 2.81). Red was not rated significantly differently from either achromatic or green (all *p*s > 0.05), and the achromatic, and green backgrounds did not differ significantly from each other (*p* = 0.81).

## Discussion

Results indicated that red has an affective meaning, consistent with previous findings based on the congruency effect (e.g., Gerend and Sias, 2009; Moller et al., 2009; Kuhbandner and Pekrun, 2013; Young et al., 2013). Humans are often exposed to ambiguous information in everyday life, and the results of our experiment involving the processing of ambiguous stimuli indicated that as well as potentiating target information (i.e., the congruency effect between color and a target stimulus), red can cause ambiguous information to be perceived negatively. Moreover, unlike Young et al.’s (2013), our experimental design involved the presentation of images of facial expressions embedded in colored backgrounds without any priming of color. Our investigation of the effects of the meaning conveyed by the color red therefore took place in a more constrained condition, where face and color were processed simultaneously.

Our study showed that red is negatively charged, but also indicated the positive meaning of green. Indeed, the key finding is that red prompted participants to perceive the same set of faces more negatively than green did, with the two control colors producing intermediate results. Moreover, this distinction, revealed in our implicit task, appeared to be in accord with the explicit measures on the subjective scales. Once again, our results converged strongly with previous findings indicating that green conveys positive meaning (e.g., Kuhbandner and Pekrun, 2013; Gil and Le Bigot, 2014). However, not only has the color green been studied less than red, but the findings are also far less consistent. The fact that green can sometimes appear to be positively valenced, and other times not, can be interpreted in the light of the color-in-context theory developed by Elliot et al. (2007). Although it depends on culture and context, green has been specially linked to positive meanings, and more particularly to positive meanings relating to appetite, growth, or creative performances (e.g., Akers et al., 2012; Lichtenfeld et al., 2012). Although speculative at this point, it is conceivable that the red-negative meaning is stronger than the opposite green-positive meaning because of what is called the *negative bias* in emotion research, whereby a negatively valenced stimulus signals events that could be detrimental to human wellbeing (e.g., Baumeister et al., 2001; Vaish et al., 2008). Moreover, this adaptive characteristic of color meaning can be linked to the ecological valence theory developed by Palmer and Schloss (2010), as people know which colors *look bad or good* for them.

Beyond the color effect *per se*, our study also investigated whether a negatively valenced color can enhance the processing of another type of valenced information, namely gender. For female facial expressions, the color effect was the same for all participants, with no modulation by decoder’s gender or facial emotion (neutral or surprised). In other words, when women and men saw a female target, the color-meaning association seemed to be the sole contextual feature that influenced face processing. By contrast, when women and men processed a male target, findings were substantially more complex, as color, expressed emotion, and decoder’s gender interacted with each other.

First, neutral faces elicited more negative responses than surprised faces did, implying less variation in the influence of color background on face processing for neutral expressions. In turn, given that surprised faces have a less negative meaning than neutral faces, the responses they elicited were influenced more by the negatively valenced color backgrounds. Indeed, there is some debate as to just how neutral neutral facial expressions are, with neutral faces generally being regarded as more negative than neutral (e.g., Palermo and Coltheart, 2004; Lee et al., 2008), consistent with our finding. The negativity of the neutral faces in the present study seemed to trigger a congruency effect, as there was a difference in the magnitude of the effect between congruent conditions (i.e., male faces with a neutral expression) and incongruent conditions (i.e., male faces expressing surprise).

Second, consistent with the literature, our findings support the notion of a valenced gender effect, as male faces were judged more negatively when the participants were female (e.g., Hess et al., 1997; Biele and Grabowska, 2006).

Taken together, these findings for male faces–statistically represented in a second-order interaction–suggest that the valenced-color effect for red can either potentiate the negativity or attenuate the positivity of other sources of information. Nevertheless, regarding the idea of a *grounding context* for face processing, the fact that our findings were so straighforward for female faces, in contrast to those for males, suggests that multiple sources of contextual information combine to create a valence-based effect, but that there is a hierarchy of sources. It is conceivable that the target’s intrinsic characteristic (i.e., poser’s gender) has the greatest influence on face processing. This finding should have consequences for future studies, but needs more investigation. Whatever the case, our results support the notion, as discussed by Palmer et al. (2013), that color can be related to other emotional stimuli in what is called *crossmodal correspondences* (e.g., Spence, 2011). It could be inferred from our results that the color red, the information conveyed by neutral expressions, and the male gender stereotype all have an emotionally negative dimension. Caution needs to be exercised, however, with regards to possible limitations of our study, which include the nature of the emotional stimuli we contrasted (i.e., neutral and surprise faces), the limited number of colors we investigated, and population that included only students. Further explorations of emotion-color relationship may yield more information about how emotional (in) congruency with regards to color emerges, and why color has such an implicit and such powerful emotional meaning for humans.

## Conflict of Interest Statement

The authors declare that the research was conducted in the absence of any commercial or financial relationships that could be construed as a potential conflict of interest.
