# Supplementary material for: Grounding context in face processing: color, emotion, and gender
Source: Front Psychol. 2015 Mar 24;6:322. doi: 10.3389/fpsyg.2015.00322 (PMC4371586; doi:10.3389/fpsyg.2015.00322)
Supplement: Supplementary file 1 [file statistical_analyses_and_details_of_the_random_effects_structures.doc]

**Supplementary Material**

***Statistical analyses and details of the random effects structures***

Logistic and linear mixed models were used to analyze the data. This procedure was designed to fit generalized linear mixed models, also called random coefficients or multilevel/hierarchical models. Random intercepts are used to account for potential variability across analysis units (which corresponded here to participants and faces). Random slopes are used to account for analysis units that potentially differ in their sensitivity to the within-unit fixed effects (which corresponded here to the study’s IVs). The maximum random effects structure justified by the experimental design (Barr et al., 2013) was included in the model. To accommodate the dependence caused by repeated measures, initial models are parameterized with random intercepts and slopes (note: between-unit treatments require only the by-unit random intercepts), along with the covariance between the variance components. The convergence problem concerning the G matrix occurs when the variation associated with at least one of the random effects (intercept or slope) that are specified is either null or negative. The solution consists in identifying the effect(s) causing the convergence problems and removing it or them from the model (Kiernan et al., 2012). Our model featured a structure with participants and faces as separate random intercepts. The three IVs used in each analysis were color (red, achromatic, mixed green/red and green), emotion (neutral and surprised faces), and sex of participants (male vs. female). We considered a full factorial design for fixed effects and a maximal random structure justified by the experimental design (i.e., all random intercepts and only random slopes corresponding to within-participants or face IVs). It should be noted that models with only main effects and a significant interaction did not modify results. Tables 1 and 2 present the final random structure of the analyses for female and male faces, respectively. As the number of observations varied across participants, we applied the Satterthwaite correction (Keselman et al., 1999).

In order to specify the interactions, we ran multiple comparison tests for each significant result using the least-squares means (LSMEANS) option of the MIXED procedure with Bonferroni adjustment, and the error degrees of freedom were row adjusted (ADJDFE=ROW option).

Table 1. Final random structure of the analyses for female posers

| Covariance parameters | Subject | Estimate | Standard error |
| --- | --- | --- | --- |
| Emotion | Participant | .9855 | .1688 |
| Color | Participant | .07253 | .002454 |
| Intercept | Face | .5782 | .3310 |
| Emotion | Face | .7862 | .2436 |
| Sex of participants | Face | .1043 | .04213 |

Table 2. Final random structure of the analyses for male posers

| Covariance parameters | Subject | Estimate | Standard error |
| --- | --- | --- | --- |
| Intercept | Participant | .05085 | .1821 |
| Emotion | Participant | 1.0168 | .2414 |
| Color | Participant | .06925 | .002544 |
| Intercept | Face | .8650 | .3775 |
| Emotion | Face | .76743 | .2110 |
| Sex of participants | Face | .007303 | .01375 |

Barr, D. J., Levy, R., Scheepers, C., and Tily, H. J. (2013). Random effects structure for confirmatory hypothesis testing: Keep it maximal. J Mem Lang, 68(3), 255‑278.

Keselman, H. J., Algina, J., Kowalchuk, R. K., and Wolfinger, R. D. (1999). The analysis of repeated measurements: A comparison of mixed-model Satterthwaite F tests and a nonpooled adjusted degrees of freedom multivariate test. Communications in Statistics – Theory and Methods, 28, 2967‑2999. doi:10.1080/03610929908832460

Kiernan, K., Tao, J., and Gibbs, P. (2012). *Tips and strategies for mixed modeling with SAS/STAT procedures.* Paper presented at the 2012 SAS Global Forum, Orlando, FL.
